# Training Physicians in the Digital Health Era: How to Leverage the Residency Elective

**DOI:** 10.2196/46752

**Published:** 2023-07-14

**Authors:** Esther Y Hsiang, Smitha Ganeshan, Saharsh Patel, Alexandra Yurkovic, Ami Parekh

**Affiliations:** 1 Department of Medicine University of California, San Francisco San Francisco, CA United States; 2 Department of Pediatrics Stanford Medicine Palo Alto, CA United States; 3 Included Health San Francisco, CA United States

**Keywords:** digital health, care delivery innovation, physician-leader, medical training, residency education, eHealth, residency, medical education, software, elective, intern, telehealth, telemedicine

## Abstract

Digital health is an expanding field and is fundamentally changing the ways health care can be delivered to patients. Despite the changing landscape of health care delivery, medical trainees are not routinely exposed to digital health during training. In this viewpoint, we argue that thoughtfully implemented immersive elective internships with digital health organizations, including start-ups, during residency are valuable for residents, residency programs, and digital health companies. This viewpoint represents the opinions of the authors based on their experience as resident physicians working as interns within a start-up health navigation and telehealth company. First, residents were able to apply their expertise beyond the traditional clinical environment, use creativity to solve health care problems, and learn from different disciplines not typically encountered by most physicians in traditional clinical practice. Second, residency programs were able to strengthen their program’s educational offerings and better meet the needs of a heterogenous group of residents who are increasingly seeking nontraditional ways to learn more about care delivery transformation. Third, digital health companies were able to expand their clinical team and receive new insights from physicians in training. We believe that immersive elective internships for physicians in training provide opportunities for experiential learning in a fast-paced environment within a field that is rapidly evolving. By creating similar experiences for other resident physicians, residency programs and digital health companies have a key opportunity to influence future physician-leaders and health care innovators.

## Introduction

Digital health is an expanding field and promises to be a significant disruptor of health care delivery [1]. Although the use of technology in health care has been percolating for several decades, the field of digital health has exponentially grown with new medical technology, creation of health policy innovation centers, and invigorated private sector interest in health care. The global digital health market is projected to grow from US $183 billion in 2020 to US $509 billion by 2027 [2], and in the United States, digital care and telehealth have the highest compound annual growth rate among all segments of digital health [3]. This boom in digital health is fundamentally changing the ways health care can be delivered to patients.

Despite the changing landscape of health care delivery and practice, medical trainees have not traditionally been exposed to digital health during training, and residency programs have not kept pace with opportunities to train physician-innovators in digital health. Currently, digital health education in medical training appears to be sparse, and it primarily takes the form of elective curricula for medical students [4,5]. This lack of exposure during the formative years of shaping medical practice can limit awareness of the changing landscape of practicing medicine.

The inherent flexibility of using an elective internship to learn more about digital health aligns well with the ever-changing digital health care landscape. However, immersive experiential learning opportunities like this, which go beyond traditional medicine, are uncommon in residency.

Resident physicians are uniquely positioned to benefit from an immersive elective internship at this point in their medical training; they have more substantial clinical experience than medical students, but they are not yet fully ingrained in the traditional health care system’s mindset and still maintain some level of career path flexibility. In this viewpoint, we argue that immersive elective internship experiences with a digital health company should be considered by resident physicians eager for unique, hands-on learning opportunities in care delivery transformation outside of usual residency training. We share our experiences in setting up an elective internship program for resident physicians at a start-up digital health company and summarize the benefits and learnings of the experience for resident physicians, residency programs, and digital health companies.

## The Implementation and Structure of 3 Immersive Elective Internships Within a Digital Health Start-Up Company

We designed an immersive elective internship for 3 resident physicians from the University of California, San Francisco with interests in digital health and care delivery transformation. The internship involved working experientially as clinical strategy interns within a start-up telehealth and health care navigation company (Included Health). Participants included 2 internal medicine residents and 1 pediatrics resident. These 3 internships took place between April 2019 and January 2022, in 4- to 6-week rotations.

The origins of this internship program arose organically from conversations between the resident physicians and a leader in this digital health organization who is also an adjunct faculty member at University of California, San Francisco. Through sharing interests, experiences, and common goals, we found that there was an opportunity to set up an internship at this digital health organization in the clinical strategy group.

To establish the internship program, we worked closely with the leadership of the residency program and the digital health organization to develop a proposed elective structure and curricular objectives. The curricular objectives were designed to align with fulfilling core competencies outlined by Accreditation Council for Graduate Medical Education, as summarized in Table 1 [6]. Our residency program leadership worked with the digital health organization and our institution’s Office of Graduate Medical Education to enable internships structured as elective rotations in residency. Residency program graduation requirements and medical responsibilities of a hospital limited the internship to 4-6 weeks in duration.

We also made efforts to identify a dedicated internal mentor within the digital health organization for the resident physicians participating in the program. The mentor was a physician-leader in the organization who inherently understood the background, knowledge, and skills of resident physicians. This was beneficial for optimizing the learning experience and ensured that the resident physicians were efficiently deployed to well-scoped projects. The mentor met with the participating residents before each internship to shorten the onboarding learning curve and worked closely with the residents throughout the duration of the internship to guide them in their work.

All 3 internship experiences were designed to meet the outlined curricular objectives. The specific content of each internship experience was significantly shaped by the digital health organization’s strategic and operational priorities during the respective period of each individual internship. The initiatives worked on by the resident physician interns included the development of pediatric care management programs, the design of integrating pharmacy services into telehealth primary care, the analysis of clinical patterns for telehealth primary care and behavioral health services, and the clinical vetting of telehealth and hybrid primary care service competitors. This work often entailed data analysis, clinical shadowing, secondary research, stakeholder interviews, and various other tasks.

**Table 1:** Designed curricular objectives aligned with Accreditation Council for Graduate Medical Education (ACGME) Core Competencies for resident physicians interning for 4-6 weeks at a digital health start-up organization.

Objectives	ACGME Core Competencies
Deepen knowledge of patient-facing challenges in health care navigation and access to care and apply to outpatient and transition-of-care clinical practice	Medical knowledge Patient care
Learn the practice of implementing and assessing new interventions to affect downstream patient outcomes	Practice-based learning and improvement
Develop knowledge of health care navigation ecosystem and resources	Systems-based practice
Work effectively with interdisciplinary, cross-functional members and create deliverables for relevant stakeholders associated with the assigned project	Interpersonal and communication skills Professionalism

## The Value of Immersive Elective Internships During Residency

Based on our experience and supporting research, we believe there are multiple benefits of an immersive elective internship experience in digital health for residents, residency programs, and digital health organizations, including start-ups.

## Benefits and Learnings for Resident Physicians

All participating resident physicians agreed that the experience provided abundant hands-on learning in a fast-paced environment during a short time and that they were able to apply their knowledge of patient care delivery to their internship experiences. Moreover, all concurred that the internship was highly formative in influencing how they considered next steps in their careers as practicing clinicians. Voluntary qualitative feedback by participating resident physician interns highlighted the following key lessons learned by residents during the internship that can be applied to their future careers.

First, the immersive internship allowed resident physicians to recognize how physician-leaders can apply their expertise in many ways beyond the traditional clinical environment**.** Participating residents worked with physician-leaders in the organization who played various roles, ranging from setting clinical strategy to leading clinical care delivery. According to one resident, “What struck me the most was the variety of ways that physicians can lend their clinical expertise to create impact within different functions.”

Second, residents learned to collaborate with a diverse range of disciplines to solve health care problems. This experience provided the first opportunity for most residents to work closely with individuals from disciplines that are not typically encountered in the traditional clinical practice of a physician. From data scientists, product managers, designers, and engineers, residents learned about opportunities and challenges for designing solutions for patients and clinicians. Although many large health systems often employ individuals with backgrounds in these fields, physicians rarely have the opportunity to closely interact and collaborate with these disciplines on a daily basis to work toward a common goal. This immersive internship allowed physicians in training to enhance their skills in team-based professionalism and communication [6].

Third, residents were able to experience how creativity can be used to approach health care problems in new ways. Traditional training in medicine tends to emphasize the role of repetition, pattern recognition, and clinical reasoning based on a repertoire of memorized facts and knowledge. Participating resident physicians agreed that they could see how novel approaches and design thinking were employed to approach problems in health care, such as improving patient messaging and clinical workflows to be more patient-centric and user-friendly. According to one resident, “I saw more discussions asking ‘what if?’ and ‘how should we?’ rather than ‘how can we make this work within existing constraints?’ and it felt like a shift in the default mindset that I am used to.”

## Benefits and Learnings for the Residency Program

Residency programs that develop an opportunity for an immersive elective internship program at digital health start-ups may strengthen their program’s educational offerings and development of their resident physicians in several ways. First, residents may be able to gain valuable skills applicable to residency training from an immersive elective experience. A recent survey of residency program directors found that more than two-thirds of respondents believed that physicians in training can gain communication and leadership skills, organizational and team-based skills, and the ability to innovate from start-up experiences [7]. Second, an increasing number of residency program applicants are entering residency with entrepreneurial experience and interest in digital health [7,8]. Providing the opportunity for an elective internship at a digital health start-up during the course of residency training can help programs meet the interests of an emerging generation of physicians in training. Finally, offering this opportunity for cross-functional training outside of the traditional clinical setting aligns with an increasingly broader call for new training opportunities in leadership for physicians. Some argue that traditional settings are no longer sufficient for developing physician-leaders and that it is crucial for leaders to engage today’s physicians in training in experiential learning to embrace their eagerness for innovation and ultimately encourage system transformation [9-11].

## Benefits and Learnings for Digital Health Organizations

Digital health organizations, including start-ups, can benefit from a resident physician immersive internship program. Physicians in training can bring a new perspective to the company by applying their up-to-date knowledge of clinical practices, recent experiences of care delivery, and intimate knowledge of both physician and patient needs [10]. For example, one resident physician was able to provide examples in her internship for how certain patients with specific disease processes may benefit from interacting with chatbots to triage clinical concerns. Resident physician interns also provide the digital health organization with the advantage of expanded capacity to tackle specific, time-bound projects of high priority. As another example, having a resident physician involved in the design of pharmacy service integration into telemedicine-based primary care services proved beneficial by providing the perspective of a clinician who may prescribe medications in various scenarios.

## Creating Opportunities for Immersive Elective Internship Experiences During Residency

As the field of digital health is constantly evolving and rapidly changing, elective internships for physicians in training provide opportunities for immersive experiential learning in a fast-paced environment. The impact of the internship can stretch far beyond the dedicated immersive experience alone; it can influence career steps and serve as a launching point for cultivating physician-leaders who can meaningfully engage across the traditional and digital health landscapes. By creating more experiences like this for other resident physicians, residency programs and digital health organizations have a key opportunity to influence physician-leaders and health care innovators of the future.
